# Continuous Interscalene Nerve Block for a Midshaft Clavicle Fracture: An Opioid-Sparing Postoperative Analgesic Strategy

**DOI:** 10.7759/cureus.49027

**Published:** 2023-11-18

**Authors:** Daniel A Arnaut, Theodis Maltbia, Hamed Sadeghipour

**Affiliations:** 1 Anesthesiology and Critical Care, Saint Louis University School of Medicine, Saint Louis, USA; 2 Anesthesiology and Critical Care, Sisters of St. Mary (SSM) Saint Louis University Hospital, Saint Louis, USA

**Keywords:** midshaft clavicle fracture, postoperative pain control, ultrasound-guided regional anesthesia, opioid-sparing analgesia, post-operative pain control, mid shaft clavicle fracture, continuous peripheral nerve block, interscalene nerve block

## Abstract

Postoperative pain after surgical clavicle fixation is difficult to treat and often responds incompletely to opioid analgesics. Unfavorable side effects and the risk of misuse of opioid analgesics make regional anesthetic techniques an attractive strategy for treating clavicular pain. Literature on continuous nerve blocks with catheter placement for more prolonged pain control for clavicle fractures is scarce, while such techniques are common for other shoulder surgeries. This case report presents a successful continuous interscalene brachial plexus block (ISB) after surgical fixation of a midshaft clavicle fracture. The patient was discharged home on the day of the operation with a portable pump, which provided a local anesthetic infusion for five days postoperatively. The patient was very satisfied with her pain control and only required one dose of oral opioid analgesic postoperatively.

## Introduction

Clavicle fractures represent approximately 2.6% of reported fractures and 44% of fractures involving the shoulder girdle. Midshaft clavicle fractures are the most common, representing approximately 75% of clavicle fractures [1]. When clavicle fractures require open reduction and internal fixation (ORIF), a general anesthetic (GA) approach is typically chosen; however, clavicle fixation with only regional anesthesia has been reported and appears to be a feasible option for some patients. Due to the complex and variable innervation of the clavicle [2], regional anesthetic techniques for clavicle fixation are difficult to achieve with one block. As a result, several peripheral nerve blocks and block combinations have been trialed [1], and much debate still exists regarding the optimal approach.

Most of the research into regional anesthesia for clavicle fractures, with and without GA, describes single-shot blocks for perioperative anesthesia. In general, postoperative pain data are limited to the day of surgery [1]. There are limited data regarding the management of clavicle ORIF pain with perineural catheter placement. In this case report, we present an 18-year-old female with a closed displaced midshaft clavicle fracture who underwent clavicle ORIF under GA. Postoperatively, she received an interscalene brachial plexus block (ISB) with nerve catheter placement for postoperative anesthetic infusion for five days. This case demonstrates an effective opioid-sparing postoperative analgesic strategy following surgical fixation of midshaft clavicle fractures.

## Case presentation

**Figure 1:**
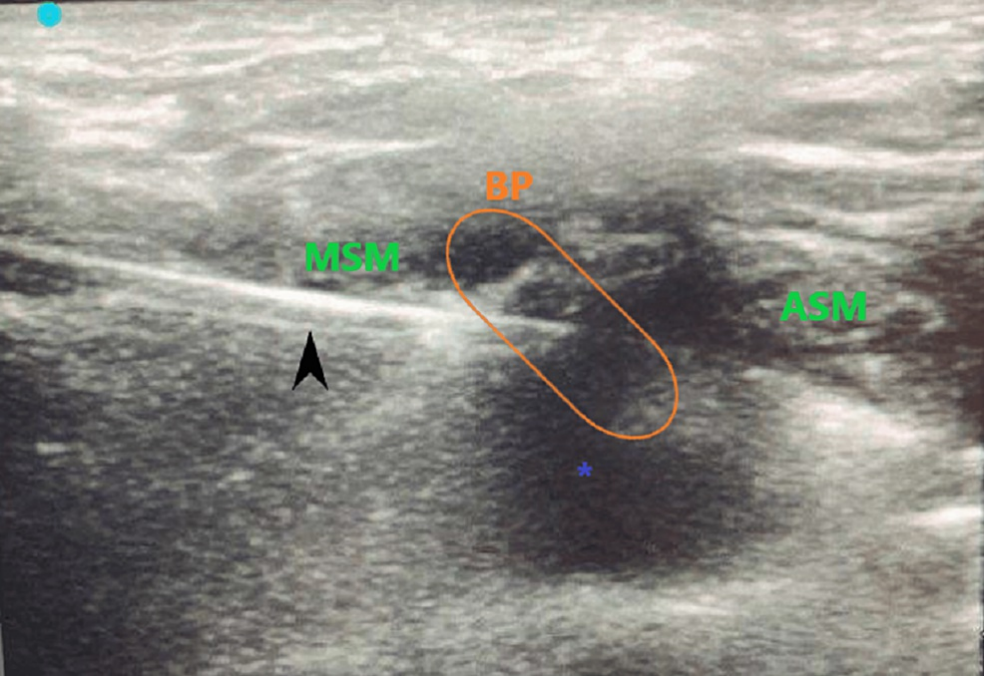
Tuohy needle (18 gauge) with perineural spread of the anesthetic BP: brachial plexus; ASM: anterior scalene muscle; MSM: middle scalene muscle; black arrow: needle; *: local anesthetic spread

An 18-year-old, 78.4 kg female (American Society of Anesthesiologists (ASA) grade 2) with no medical history was scheduled for ORIF of a closed displaced midshaft right clavicle fracture after non-union with conservative therapy. Prior to surgery, she consented to postoperative ISB with nerve catheter placement. The patient was taken to the operating room and underwent ORIF of her right clavicle under GA with no complications. She was extubated at the end of the procedure and transferred to the post-anesthesia care unit (PACU) for postoperative monitoring. There, the regional anesthesia team prepared the patient for the nerve block. She was positioned with the head of the bed up 30 degrees and with her head turned approximately 45 degrees towards the left. After disinfection of the skin, approximately 5 mL of lidocaine 1% was used for infiltration of the skin and subcutaneous tissues overlying the interscalene groove. An 18G Tuohy needle was guided towards the brachial plexus under ultrasound guidance using an in-plane approach. Aspiration was performed to confirm that the needle tip was extravascular, and 15 mL of 0.25% bupivacaine with epinephrine (1:200,000), as well as 4 mg dexamethasone, were used for the single-shot block without any complications. Intermittent repositioning and hydro dissection were utilized to ensure adequate distribution of anesthetic. The perineural spread of the anesthetic was visualized (Figure 1).

**Figure 2:**
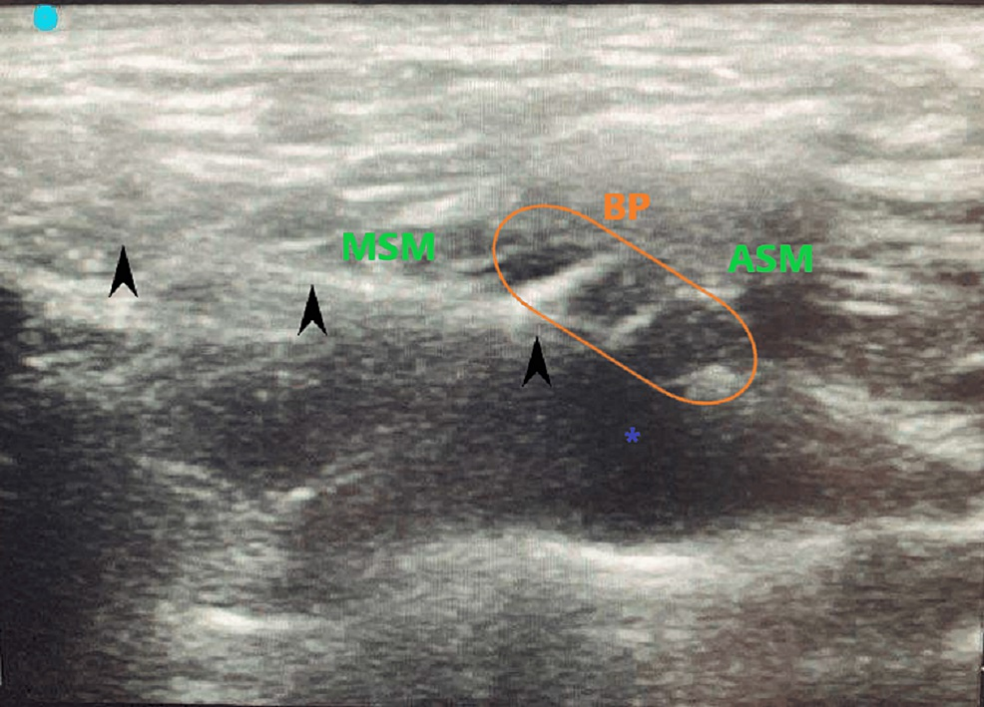
Nerve catheter (20 gauge) in place after removal of the needle BP: brachial plexus; ASM: anterior scalene muscle; MSM: middle scalene muscle; black arrow: catheter; *: local anesthetic spread

Then, a 20G nerve catheter (closed-tip Contiplex® Echo catheter, Braun Medical, Bethlehem, PA) was passed through the needle, 3 cm past the needle tip, and the needle was removed, leaving the catheter in place. There were no complications, and the catheter placement was confirmed with ultrasound (Figure 2).

The patient was satisfied with the pain control provided by the nerve block prior to discharge.

The patient was discharged with 400 mL of 0.2% ropivacaine in a disposable pump (ON-Q® PainBuster® pump, Avonas Medical, Alpharetta, GA) with the rate set at 4 mL/hr. The patient was instructed to increase the basal rate of the pump if her pain score worsened and to only use opioids if the catheter was not providing pain relief at its maximum rate. On her ride home on postoperative day (POD) #0, the pump rate was briefly increased to 8 mL/hr, and she took one oxycodone/acetaminophen (5 mg/325 mg) tablet for 7/10 pain. Later that night, she decreased the rate to 6 mL/hr, where it remained until catheter removal on POD #5. The patient was called each day while the nerve catheter was in place. From POD #1 to POD #5, she did not take any opioids, and her pain score never got above 3/10. She also did not have to increase her infusion rate for the remainder of the continuous block. The patient reported satisfaction with the level of pain control at home. On POD #5, the patient removed the catheter at home as instructed, and the catheter tip remained intact.

## Discussion

With the increased awareness of the dangers of opioids, regional anesthetic techniques are increasingly important to understand as an alternative analgesic strategy. At present, pain from clavicle fractures and surgical repair is not routinely treated with a continuous nerve block. This may be, in part, due to the complex and variable innervation of the clavicle. Practitioners may perhaps be hesitant to place a nerve catheter due to the concern of incomplete pain coverage with a single catheter placement. An ISB with perineural catheter placement has been shown to significantly improve postoperative pain after other shoulder surgeries [3,4] but has not been demonstrated for clavicle ORIF. One case report describes continuous nerve block for postoperative pain in clavicle ORIF but required multiple catheter placements involving the C5 nerve root and a superficial cervical plexus block to achieve adequate pain relief [5]. Our case is unique in that we achieved adequate postoperative analgesia for clavicle surgery with continuous ISB alone.

Based on current literature, the most commonly utilized regional anesthesia for clavicle fracture ORIF is an ISB combined with an intermediate cervical plexus block (ICPB), especially when proceeding without GA. This combination seems to be the most reliable for complete pain coverage intraoperatively [1,6-8]. However, despite the feasibility of clavicle ORIF with regional anesthesia only, many centers prefer to perform clavicle surgery under GA for maximum patient comfort intraoperatively. With GA, the use of both ISB and ICPB may be unnecessary. One study compared ICPB alone versus a combination of ISB plus ICPB in patients undergoing surgical clavicle fixation under GA. They found that ICPB was non-inferior to the combined ISB plus ICPB for perioperative analgesia, suggesting that ISB was redundant under these circumstances [9]. However, without GA, both ISB and ICPB alone are insufficient as a sole anesthetic for clavicle fracture ORIF, requiring additional blockade or conversion to GA intraoperatively [5,6].

Despite ISB being insufficient as a sole intraoperative anesthetic for clavicle surgery [6], some studies show effective opioid-sparing analgesia perioperatively when only a single-shot ISB is used with GA [10]. Knowing this, the analgesic efficacy of an ISB for clavicle ORIF can be extended by placing a nerve catheter, as has been demonstrated for other shoulder surgeries [3,4]. With prolonged infusion, an ISB catheter can theoretically provide more complete coverage as compared to a single-shot block for clavicle pain due to the local spread of anesthetic, as described in the next paragraph.

There is much uncertainty regarding the relative importance of the nociceptive innervation of the clavicle. A cadaveric dissection revealed the contributing nerves [2], which guides the discussion regarding anesthetic blockade of clavicle pain. The cephalad and ventral aspects of the clavicle as well as the sternoclavicular joint are innervated by the supraclavicular nerve, which is composed of the C3-C4 nerve roots. The caudal and dorsal aspects of the middle and medial thirds of the clavicle are innervated by the subclavian nerve, which is composed of the C5-C6 nerve roots. Finally, the caudal and dorsal aspects of the middle and lateral thirds of the clavicle are innervated by the lateral pectoral nerve, which is composed of the C5-C7 nerve roots [2]. The ISB targets the C5-C7 nerve roots directly with a local anesthetic injected around these nerve roots. In addition, branches of the supraclavicular nerve (C3-C4) are anesthetized due to the superficial and proximal spread of anesthetic [11]. Therefore, with a sufficient local anesthetic dose, especially with prolonged infusion, pain from the midshaft clavicle ORIF may be blocked with an ISB alone.

Every nerve block has its own set of risks, and multiple nerve blocks on the same patient increase the chances of complications. Both ISB and cervical plexus block risks include inadvertent intravascular puncture, intravascular injection of anesthetic, and nerve damage. These risks can be mitigated with the use of ultrasound guidance. Other shared complications include infection, bleeding/hematoma, and Horner’s syndrome [12,13]. Cervical plexus block can cause dysfunction of the recurrent laryngeal nerve and phrenic nerve (although the latter is far more common with ISB [10]). An ISB is very likely to cause ipsilateral phrenic nerve dysfunction for the duration of the block, decreasing respiratory function by up to 25% [12]. Therefore, it is important to consider the patient’s respiratory function before performing this block. In addition, inadvertent recurrent laryngeal nerve palsy can cause airway obstruction, especially in patients with existing contralateral vocal cord paralysis [12].

Other limitations of continuous nerve blocks include patient discomfort and limited mobility postoperatively, as well as increased cost to the patient compared to oral analgesics. The use of multiple nerve catheters and pumps may further increase discomfort and cost. Therefore, if it is feasible to achieve adequate pain control with a single nerve block and catheter, such a strategy should be used.

## Conclusions

This case exhibits a successful use of an opioid-sparing regional anesthetic for postoperative pain in patients with midshaft clavicle fractures. For the right patient, an ultrasound-guided ISB with catheter placement can provide safe and highly effective analgesia for several days following clavicle fixation, leading to high patient satisfaction. More clinical trials are needed to evaluate the efficacy of this approach, as well as to quantify the rate and severity of complications to further guide the practitioner’s risk-benefit consideration for this procedure.

## References

[1] Lee CC, Beh ZY, Lua CB, Peng K, Fathil SM, Hou JD, Lin JA (2022). Regional anesthetic and analgesic techniques for clavicle fractures and clavicle surgeries: part 1—a scoping review. Healthcare (Basel).

[2] Leurcharusmee P, Maikong N, Kantakam P, Navic P, Mahakkanukrauh P, Tran D (2021). Innervation of the clavicle: a cadaveric investigation. Reg Anesth Pain Med.

[3] Klein SM, Grant SA, Greengrass RA (2000). Interscalene brachial plexus block with a continuous catheter insertion system and a disposable infusion pump. Anesth Analg.

[4] Mariano ER, Loland VJ, Ilfeld BM (2009). Interscalene perineural catheter placement using an ultrasound-guided posterior approach. Reg Anesth Pain Med.

[5] Kline JP (2013). Ultrasound-guided placement of combined superficial cervical plexus and selective C5 nerve root catheters: a novel approach to treating distal clavicle surgical pain. AANA J.

[6] Gupta N, Gupta V, Kumar G, Gupta V, Gupta DK (2019). Comparative evaluation of efficacy of interscalene block vs. interscalene block and superficial cervical plexus block for fixation of clavicular fractures. Int J Contemp Med Res.

[7] Ryan DJ, Iofin N, Furgiuele D, Johnson J, Egol K (2021). Regional anesthesia for clavicle fracture surgery is safe and effective. J Shoulder Elbow Surg.

[8] Balaban O, Dülgeroğlu TC, Aydın T (2018). Ultrasound-guided combined interscalene-cervical plexus block for surgical anesthesia in clavicular fractures: a retrospective observational study. Anesthesiol Res Pract.

[9] Abdelghany MS, Ahmed SA, Afandy ME (2021). Superficial cervical plexus block alone or combined with interscalene brachial plexus block in surgery for clavicle fractures: a randomized clinical trial. Minerva Anestesiol.

[10] Olofsson M, Taffé P, Kirkham KR, Vauclair F, Morin B, Albrecht E (2020). Interscalene brachial plexus block for surgical repair of clavicle fracture: a matched case-controlled study. BMC Anesthesiol.

[11] Gautier PE, Vandepitte C, Gadsden J (2023). Ultrasound-guided interscalene brachial plexus nerve block. https://www.nysora.com/techniques/upper-extremity/intescalene/ultrasound-guided-interscalene-brachial-plexus-block/.

[12] Zisquit J, Nedeff N (2023). Interscalene Block. https://www.ncbi.nlm.nih.gov/books/NBK519491/.

[13] Hipskind JE, Ahmed AA (2023). Cervical Plexus Block. https://www.ncbi.nlm.nih.gov/books/NBK557382/.

